# Extending Tests of Hardy–Weinberg Equilibrium to Structured Populations

**DOI:** 10.1534/genetics.119.302370

**Published:** 2019-09-19

**Authors:** Wei Hao, John D. Storey

**Affiliations:** Lewis-Sigler Institute for Integrative Genomics, Princeton University, New Jersey 08544

**Keywords:** admixed, admixture, Chi-square test, HWE, population genetics, quality control, random mating, significance test

## Abstract

Testing for Hardy–Weinberg equilibrium (HWE) is an important component in almost all analyses of population genetic data. Genetic markers that violate HWE are often treated as special cases; for example, they may be flagged as possible genotyping errors, or they may be investigated more closely for evolutionary signatures of interest. The presence of population structure is one reason why genetic markers may fail a test of HWE. This is problematic because almost all natural populations studied in the modern setting show some degree of structure. Therefore, it is important to be able to detect deviations from HWE for reasons other than structure. To this end, we extend statistical tests of HWE to allow for population structure, which we call a test of “structural HWE.” Additionally, our new test allows one to automatically choose tuning parameters and identify accurate models of structure. We demonstrate our approach on several important studies, provide theoretical justification for the test, and present empirical evidence for its utility. We anticipate the proposed test will be useful in a broad range of analyses of genome-wide population genetic data.

## Abstract (precis)

Testing for Hardy-Weinberg Equilibrium (HWE) is an important component in almost all analyses of population genetic data. Genetic markers that violate HWE are often treated as special cases; for example, they may be flagged as possible...

Hardy–Weinberg equilibrium (HWE) is a general and far-reaching principle in population genetics that is incorporated into a wide range of applications. In evolutionary terms, HWE says that for a population meeting certain conditions, the genotype frequencies of a genetic locus can be expressed in terms of the allele frequencies. The equilibrium portion of HWE comes from the fact that even if this relation does not hold in the initial state of a population, one generation of random mating guarantees that HWE will hold. An equivalent way to frame HWE is in probabilistic terms, where the relationship between genotype frequencies as a function of the allele frequency is a result of a Binomial distribution parameterized by the allele frequency for diallelic markers (or the Multinomial for multiallelic markers). The genotype of a randomly sampled individual is then determined by a draw from the Binomial distribution. Tests for HWE in practice usually involve verifying the Binomial distribution of the genotypes in terms of allele frequencies.

These simple statements about HWE motivate why testing for HWE is a common preliminary step in a variety of genomic analyses—indeed, HWE serves as a data-quality check or model-assumptions check since it is expected to approximately hold for most markers (Gillespie 2004). In studies such as genome-wide association studies (GWAS), HWE is treated as a baseline for quality control in outcrossing species, where markers deviating strongly from HWE are filtered out as likely genotyping errors (Yu *et al.* 2009; Anderson *et al.* 2010; Winkler *et al.* 2014). Further, the probabilistic statement of HWE where genotypes can be modeled using the Binomial distribution serves as the basis for many statistical methods. For instance, HWE serves as an assumption in models of population structure (Pritchard *et al.* 2000; Patterson *et al.* 2006), the calculation of genetic relationship matrices (Yang *et al.* 2011), and tests for genetic association (Price *et al.* 2006). Even when HWE is not explicitly stated to be an assumption, many common statistical genetic operations use the Binomial form of HWE, for example, scaling by the standard deviation of the Binomial in terms of allele frequencies before performing a principal components analysis (PCA) or forming a genetic-relatedness matrix.

While the broad importance of HWE to genetics is clear, it is nonetheless the case that the conditions necessary for HWE are restrictive, especially in its requirement that there is no population structure present. Considering a probabilistic approach, HWE treats observations at a marker to be independent and identically distributed, *i.e.*, completely homogeneous with no structure. Population structure is ubiquitous in human populations (Novembre and Peter 2016) and therefore they are likely to violate the no-structure assumption of HWE. This typically results in the appearance that the large proportions of markers deviate from HWE, obfuscating the important deviations such as those resulting from genotyping error or evolutionary selection.

These limitations are evident in how practitioners apply HWE to human genetic data. One approach is to test for HWE separately within subsets of the samples where there is less population structure. Test results are then aggregated at each marker, and some criteria accounting for the separate tests are applied to determine whether HWE is violated. This often appears in studies where there are known population labels for the samples (for example, Li *et al.* 2008; Coop *et al.* 2009). Another approach is to reject HWE based on a very conservative *P*-value threshold which can vary between studies. For instance, in Gormley *et al.* (2016), a meta-analysis study of 22 separate GWAS, the HWE *P*-value threshold used in each individual GWAS ranged from $10^{-20}$ to $10^{-3}$. The goal of these approaches is to reduce the number of markers violating HWE to ensure that genotyping errors are removed.

A data-driven approach was proposed in Sha and Zhang (2011), where they calculated principal components from genotype data and then performed a logistic regression-based, goodness-of-fit test with the principal components as covariates. Although this has some basic conceptual connections to the approach proposed here, our work presents an overall framework for HWE in structured populations and addresses several technical issues: (i) the principal components are calculated on the observed genotype scale, but the logistic regression is on the canonical link scale, making this model fit problematic (Hao *et al.* 2016); (ii) overfitting occurs from estimating principal components and then testing a model fit on the same data, leading to inflated levels of statistically significant departure from HWE (Chung and Storey 2015); and (iii) there is no method for identifying the number of principal components to be used (the authors suggest using 10).

We propose a procedure for testing for HWE that allows for population structure, called the “structural HWE” (sHWE) test. We address the limitations of existing HWE methods by extending the probabilistic model to allow for heterogeneity in the samples, *i.e.*, by modeling the genotypes at a marker using individual-specific allele frequencies that account for structure. Individual-specific allele frequencies are the most general parameterization of structure in that there is a unique allele frequency for each marker and individual combination, and common models of population structure can be formulated in this way, including the often-used admixture models. We discuss specific parameterizations of this model in *Methods*. Like current methods for testing for HWE, our proposed test of sHWE can be applied on a marker-to-marker basis to determine which markers violate HWE, with allowances for population structure. Further, the genome-wide joint distribution of sHWE *P*-values can be used to assess a global goodness of fit of the model of population structure. This allows us to choose optimal values of tuning parameters such as the latent dimensionality of a model or the number of admixed ancestral populations. Lastly, the assumptions of the sHWE test satisfy the conditions needed for association testing while controlling for population structure (Song *et al.* 2015).

To illustrate the flexibility of and to motivate the sHWE procedure, we analyzed single nucleotide polymorphism (SNP) genotypes from the 1000 Genomes Project (TGP) (1000 Genomes Project Consortium *et al.* 2010, 2015). The TGP data exhibit population structure in two challenging ways: first, samples were taken from populations on a global scale (including samples originally in the HapMap project) and, second, some samples such as the Hispanic Latin American populations are known to have undergone recent admixture (Bryc *et al.* 2010; Thornton *et al.* 2012; Moreno-Estrada *et al.* 2014). To model population structure, we used the logistic factor analysis (LFA) method (Hao *et al.* 2016), which uses *K* latent variables to account for structure. Increasing *K* captures progressively more of the population structure.

Figure 1 shows *P*-value histograms from this analysis, where each histogram comprises *P*-values resulting from testing for HWE or sHWE on all markers simultaneously. A traditional test for HWE is heavily skewed toward zero, indicating that the vast majority of SNPs would be found to deviate from HWE due to the presence of population structure. Fitting the LFA model with K=3 partially accounts for the population structure, and the sHWE *P*-values have a smaller peak at zero and skew less toward zero than the uncorrected HWE *P*-values. We show in *Results* that K=12 is the optimal value for the TGP data set. For K=12, the vast majority of genome-wide *P*-values are Uniform(0, 1) distributed. This is the distribution of*P*-values when sHWE holds, and it indicates that population structure has been accounted for in our model, with the exception of the small number of SNPs found to be out of sHWE and thus also out of HWE. Since the LFA fit with K=12 optimally accounts for population structure and the sHWE test incorporates population structure into the procedure, these deviations from sHWE can be attributed to technology errors or evolutionary effects.

**Figure 1:**
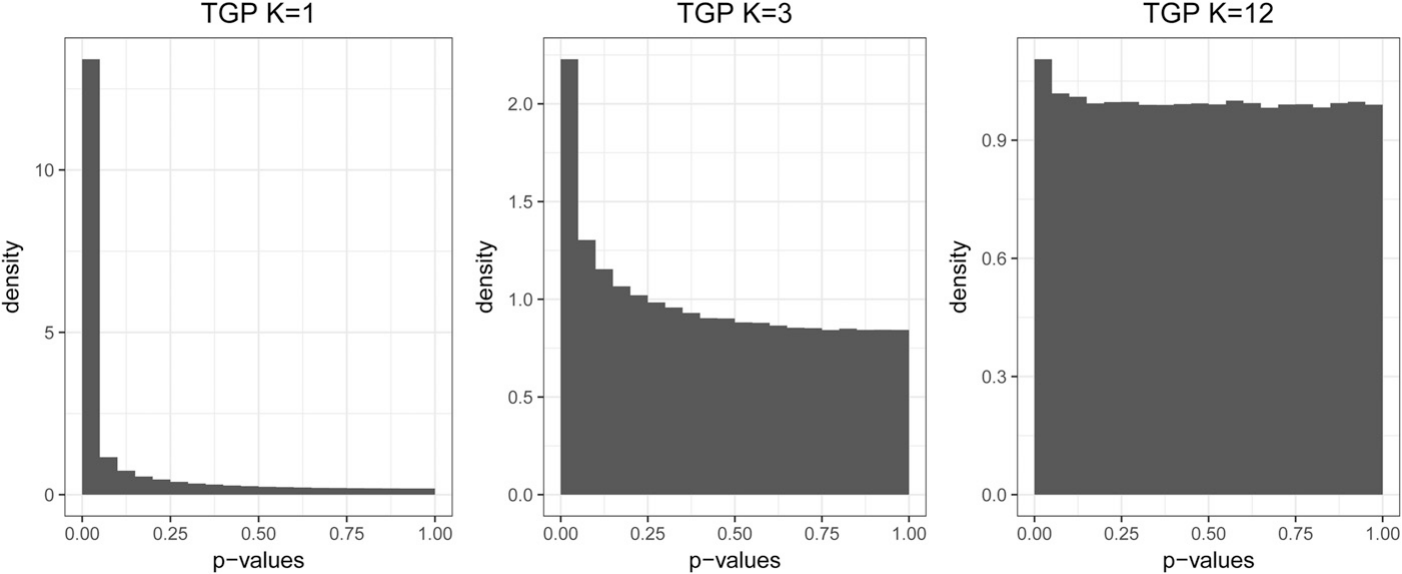
A proof of concept of the sHWE procedure. We fit the LFA model of structure (Hao *et al.* 2016) to the TGP data set and varied *K*, which is the number of latent factors to account for population structure. The left-most panel depicts a histogram of genome-wide *P*-values for a traditional test of HWE, which is equivalent to using the sHWE test with a population structure model of dimensionality K=1. The histogram is heavily skewed toward zero, showing that most SNPs would be identified as deviating from HWE. The middle panel depicts sHWE test *P*-values for K=3, which partially accounts for the population structure. As a result, there is less skew toward zero, and the large *P*-values (*i.e.*, >0.75) are Uniform distributed which indicates that some SNPs are in sHWE. The right-most panel depicts sHWE test *P*-values for K=12, the empirically optimal value, which best accounts for population structure in the data set. The SNPs concentrated at the peak near zero are found to be deviated from sHWE, indicating that they violate HWE for reasons other than population structure.

The sHWE test is performed by fitting a model of population structure that parameterizes allele frequencies for each individual and SNP pair. Then, we simulate null genotyping data sets that preserve the observed population structure where sHWE holds. Finally, we calculate a test statistic that measures deviation from sHWE for the observed and null data sets, and *P*-values are computed. The algorithm is shown in Figure 2. We demonstrate our method on several publicly available global human data sets: the Human Genome Diversity Project (HGDP) (Cann *et al.* 2002), the 1000 Genomes Project (TGP) (1000 Genomes Project Consortium *et al.* 2010, 2015), and a data set genotyped using the Affymetrix Human Origins (HO) chip (Lazaridis *et al.* 2014). We first analyze these data sets independently, showing how the sHWE procedure allows one to choose the dimensionality of the population structure model. Then, we compared SNPs that are misspecified with respect to the population structure model between data sets and technologies, showing that the sHWE procedure identifies SNPs affected by genotyping errors and that results are replicable between data sets.

**Figure 2:**
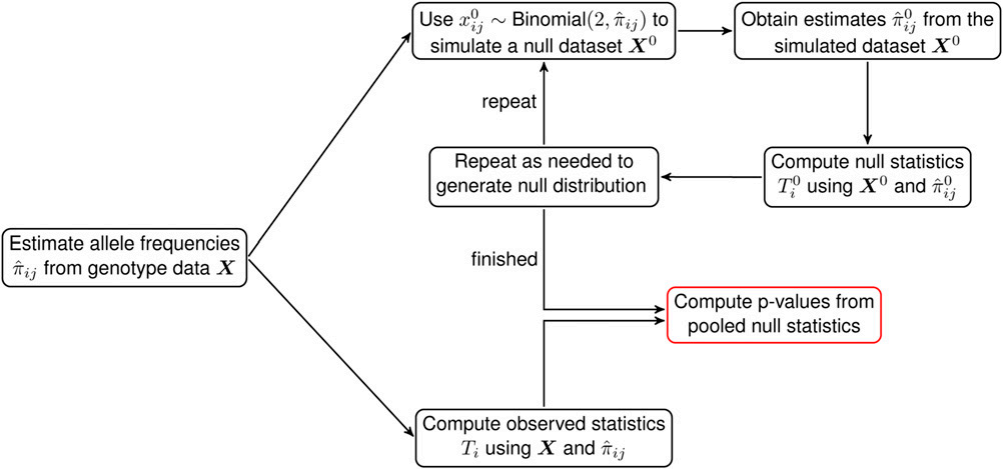
The sHWE testing procedure as a schematic. Using the genotype matrix ***X***, we first fit a model of population structure to estimate ${\hat{\pi }}_{ij}.$ The values of ${\hat{\pi }}_{ij}$ are used to simulate null data sets incorporating the sHWE assumptions. We compute sHWE test statistics for both observed and simulated null data sets and compute *P*-values by comparing the values of the observed test statistics and the pooled null test statistics.

## Methods

We first introduce the globally sampled, human genome-wide genotyping data sets used in this analysis. Then, we show how the probabilistic interpretation of HWE can be extended to the sHWE test by incorporating the most general representation of population structure. We discuss a few ways to parameterize population structure and consider how the sHWE test behaves when all parameters are known. In addition, we show how to implement the sHWE test in practice using simulated empirical null distributions based on genome-wide genotyping data. Finally, we discuss how the sHWE procedure can be used to validate and tune models of population structure in addition to the standard application of marker quality control.

### Data sets

We used genome-wide genotyping data from three publicly available sources, each of which performs global sampling of humans.

#### HGDP:

This study sampled globally from 51 populations (Cann *et al.* 2002). We filter for related individuals using the “H952” subset from Rosenberg (2006). Genotypes were filtered with minimum allele frequency of 0.05 and minimum genotype completeness of 0.995. The dimensions of this data set are 940 individuals and 550,303 SNPs. The data are available at http://www.hagsc.org/hgdp/files.html.

#### HO:

This study sampled globally from 147 populations (Lazaridis *et al.* 2014). These samples were genotyped on the Affymetrix HO array, which was specially designed for population genetics applications. We used the publicly available portion of the data set. Genotypes were filtered with minimum allele frequency of 0.05 and minimum genotype completeness of 0.99. After filtering the data set for ancient and nonhuman samples, we are left with 372,446 SNPs and 2,251 individuals. The data are available from the Reich laboratory Web site: http://genetics.med.harvard.edu/reich/Reich_Lab/Datasets.html.

#### TGP:

This study analyzed genome sequence diversity in humans through whole-genome sequencing (1000 Genomes Project Consortium *et al.* 2010, 2015). They also provide SNP chip genotyping on the Illumina Omni platform for the phase-3 release. Genotypes were filtered with minimum allele frequency of 0.05 and minimum genotype completeness of 0.99. After removing related individuals, the data set consists of 1815 individuals and 1,229,310 SNPs. The data are available at ftp://ftp.1000genomes.ebi.ac.uk/vol1/ftp/release/20130502/supporting/hd_genotype_chip/.

Further, we generated two additional data sets from TGP for the purpose of comparing individuals genotyped on different technologies: one from variants called from sequencing data (the TGP phase-3 variant calls available at ftp://ftp.1000genomes.ebi.ac.uk/vol1/ftp/release/20130502/) and the other a version of the genotyping chip data described above. Both data sets were designed to maximally overlap. We filtered both data sets to share the same unrelated individuals (1683 individuals total). We used a subset of 1,224,056 of the SNPs from the genotyping chip data overlapping with the variant calls. The variant calls were designed to include as many SNPs from the chip data as possible, as well as additional SNPs that were least 5 kbp apart. This resulted in a subset of 1,306,465 SNPs from the variant calls.

### Traditional HWE as a probability model

Typically, population genetic assumptions such as infinite population size, random mating, no selection, no mutation, and no migration (among others) are assumed as the starting point for HWE. At a particular locus with alleles *A* and *B* and allele frequencies *p* (corresponding to allele *B*) and q=1−p (corresponding to allele *A*), HWE states that after one generation of random mating, the genotype frequencies of AA,AB, and BB are $q^{2},2pq,$ and $p^{2},$ respectively. The allele frequencies and genotype frequencies then remain at these values for all further generations. HWE can be viewed as a probabilistic model if we consider *B* to be the reference allele and code the genotypes as 0, 1, and 2, corresponding to AA, AB, and BB, respectively. The genotype for each individual at this locus is modeled under HWE as an independent draw from a Binomial(2,p) distribution. The relationship between genotype frequency and allele frequency follows directly from this distributional assumption.

Common tests for HWE such as Pearson $\chi^{2}$ test for goodness of fit or Fisher’s exact test (Wigginton *et al.* 2005) check for whether the observed genotype counts are compatible with draws from a Binomial distribution using the observed allele frequency as the probability of success. Many of the population-genetics assumptions are related to the statistical assumption that alleles and individuals can be treated as independent and identically distributed. In human data sets, the presence of population structure means this assumption is violated, resulting in a deviation from HWE. We will directly account for population structure by parameterizing it in a way that is compatible with a Binomial model of genotypes so that this probabilistic model of HWE can be tested in the presence of structure.

### Models of population structure

Consider a data set consisting of *m* diallelic SNP genotypes (coded as 0, 1, and 2 copies of the reference allele) and *n* individuals. For current GWAS, *m* is often on the order of millions while *n* is in the tens of thousands. We will aggregate the data into a genotype matrix ***X*** with dimensions *m* by *n*, and choose indices such that $x_{ij}$ corresponds to the *i*-th SNP and *j*-th individual in ***X***.

For complete generality, let us allow each individual and SNP pair to have its own reference allele frequency $\pi_{ij};$ this permits a flexible parameterization so that it is possible that each individual is effectively drawn from its individual-specific population such as from an admixture model (Hao *et al.* 2016). We can aggregate the $\pi_{ij}$ into a m×n matrix ***F*** whose (i,j) element is $\pi_{ij}$ and $F=\frac{1}{2}E\left[X\right].$ This represents the most general way to probabilistically represent population structure, as there are as many parameters as there are individual and SNP pairs. Models of population structure typically parameterize $\pi_{ij}$ with constraints, so that fewer parameters are needed. Using more sophisticated parameterizations of $\pi_{ij}$ will allow us to relax the statistical assumption that the individuals are all identically distributed.

We will now summarize several special cases of the above general parameterization, where there are constraints on the $\pi_{ij}$ values. In all cases, these models assume that the genotypes are generated independently according to $x_{ij}∼\text{Binomial}\left(2,\pi_{ij}\right).$ The simplest parameterization of $\pi_{ij}$ is that of a population in HWE with no structure, where all $\pi_{ij}=p_{i}$ and $p_{i}$ is the observed allele frequency at SNP *i*. In a model with nonoverlapping, independently evolving subpopulations, there are *K* subpopulations and $\pi_{ij}$ is the allele frequency of SNP *i* for the subpopulation of which individual *j* is a member. In an admixture model of population structure (Pritchard *et al.* 2000; Alexander *et al.* 2009), there are *K* ancestral populations, and the relevant model parameters are $q_{j}$, the *K*-vector of admixture proportions for individual *j*, and $p_{i}$, the *K*-vector of allele frequencies for SNP *i*. Then, $\pi_{ij}$ is the weighted sum of these parameters, $\pi_{ij}=\sum_{k=1}^{K}p_{ik}q_{kj}.$ In spatial models of population structure (Wasser *et al.* 2004; Corander *et al.* 2008), $\pi_{ij}$ is explicitly a smooth function of the geographical coordinates of each individual.

In this article, we focus on two approaches from Hao *et al.* (2016): LFA and truncated PCA. These methods are both computationally efficient and were shown to outperform existing methods for estimating ***F***. They directly model $\pi_{ij}$ using low-dimensional factorizations, and are accurate and computational efficient on large data sets (Hao *et al.* 2016). We primarily use the LFA method, which models $\pi_{ij}$ using its canonical parameterization, $\text{logit}\left(\pi_{ij}\right)=\text{log}\left(\pi_{ij}/\left(1-\pi_{ij}\right)\right).$ Population structure is captured by factorizing the logit transformation of ***F***: logit(F)=AH, where ***A*** is an *m* × *K* matrix and ***H*** is a *K* × *n* matrix. The columns of ***H*** represent population structure for each individual, while the rows of ***A*** are the way population structure is manifested in each SNP. We also show results for the PCA approach to estimating $\pi_{ij}$.

The truncated PCA approach uses the fact that, under the Binomial model relating $x_{ij}$ and $\pi_{ij},$ $E\left[x_{ij}\right]=2\pi_{ij}.$ The estimates of $\pi_{ij}$ are formed by projecting ***X*** onto first *K* principal components of ***X*** and scaling the projection by a factor of 1/2. Some of the values for ${\hat{\pi }}_{ij}$ may be outside of the interval [0,1], in which case they are replaced by 1/(2n) or 1−1/(2n), which corresponds to the allele frequency for having only one copy of an allele. In addition to these two methods, we also demonstrate our proposed test on the ADMIXTURE (Alexander *et al.* 2009) method for modeling population structure through the probabilistic admixture model described above.

All of the models of population structure summarized here involved a tuning parameter, such as the number of ancestral populations *K*, a smoothing parameter in the spatial model, or the number of latent factors *K*. Our proposed method will introduce a way to automatically choose the value of these tuning parameters by considering the goodness of fit of the model of structure to the data.

### A structural test for HWE

Using the individual-specific allele frequencies $\pi_{ij}$ offers a general framework for extending tests of HWE to allow for structure. To this end, we will derive a test for sHWE by extending the derivation of the standard Pearson $\chi^{2}$ statistic.

The data for a single marker can be summarized using the genotype counts for a SNP, where $N^{\left(0\right)},N^{\left(1\right)},N^{\left(2\right)}$ are the number of observed 0, 1, and 2 genotypes, respectively (written as $N^{\left(G\right)}$ for G∈{0,1,2}). We put the genotype variable in parentheses and as a superscript to distinguish it from the indices for matrices and vectors. We will introduce a new test statistic that is to be calculated for each SNP. Thus, consider a fixed SNP and drop the corresponding subscript *i*. This leaves us with the vector of genotypes $x=\left(x_{1},x_{2},\ldots ,x_{n}\right)$ and a vector of allele frequencies $\pi =\left(\pi_{1},\pi_{2},\ldots ,\pi_{n}\right).$ The test of sHWE performs a hypothesis test on the distribution of the genotype data as follows:

$$\begin{array}{c} H_{0}:x_{j}∼\text{Binomial}\left(2,\pi_{j}\right)\;\forall_{j}\in \left\{1,2,...,n\right\}\;\Rightarrow \text{sHWE}\;\text{holds}\\ H_{1}:\text{not}\;H_{0}\;\;\;\;\;\;\;\;\;\;\;\Rightarrow \text{sHWE}\;\text{does}\;\text{not}\;\text{hold} \end{array}$$

This hypothesis test is performed for all markers simultaneously, with the goal of identifying which markers deviate from the sHWE assumptions.

We can write the genotype counts as $N^{\left(G\right)}=\sum_{j=1}^{n}1\left(x_{j}=G\right),$ where 1(⋅) is the indicator function. We can define the quantity $p_{j}^{\left(G\right)}=E\left[1\left(x_{j}=G\right)\right]$, which depends on the genotype *G* in the following way when sHWE holds:

$$p_{j}^{\left(G\right)}=E\left[1\left(x_{j}=G\right)\right]=\{\begin{array}{cc} {\left(1-\pi_{j}\right)}^{2} & \text{if}\;G=0\\ 2\pi_{j}\left(1-\pi_{j}\right) & \text{if}\;G=1\\ {\left(\pi_{j}\right)}^{2} & \text{if}\;G=2 \end{array}.$$ (1)

This notation will allow us to consider the distribution of $N^{\left(G\right)}$ and to formulate a test where the null hypothesis is that Equation 1 holds for all *j* and the alternative is that Equation 1 does not hold for at least one *j*. It follows that $E\left[N^{\left(G\right)}\right]=\sum_{j}p_{j}^{\left(G\right)}$ and $\mathrm{Var}\left[N^{\left(G\right)}\right]=\sum_{j}p_{j}^{\left(G\right)}\left(1-p_{j}^{\left(G\right)}\right).$ We can apply the Lindeberg version of the central limit theorem (Billingsley 2012) to show that $N^{\left(G\right)}$ is asymptotically distributed as a Normal random variable with mean $\sum_{j}p_{j}^{\left(G\right)}$ and variance $\sum_{j}p_{j}^{\left(G\right)}\left(1-p_{j}^{\left(G\right)}\right).$

Now consider just two of the genotype counts as a vector of length two called $v=\left(N^{\left(0\right)},N^{\left(1\right)}\right),$ since $N^{\left(2\right)}=n-N^{\left(0\right)}-N^{\left(1\right)}.$ It is distributed bivariate Normal with mean vector $\mu =\left(\sum_{j}p_{j}^{\left(0\right)},\sum_{j}p_{j}^{\left(1\right)}\right)$ and covariance matrix Σ, where:

$$\Sigma =\left(\begin{array}{cc} \sum_{j}p_{j}^{\left(0\right)}\left(1-p_{j}^{\left(0\right)}\right) & -\sum_{j}p_{j}^{\left(0\right)}p_{j}^{\left(1\right)}\\ -\sum_{j}p_{j}^{\left(0\right)}p_{j}^{\left(1\right)} & \sum_{j}p_{j}^{\left(1\right)}\left(1-p_{j}^{\left(1\right)}\right) \end{array}\right).$$

Thus, the quadratic form

$$T={\left(v-\mu \right)}^{T}\Sigma^{-1}\left(v-\mu \right)$$

$$={\left(v-\mu \right)}^{T}{\left(\begin{array}{cc} \sum_{j}p_{j}^{\left(0\right)}\left(1-p_{j}^{\left(0\right)}\right) & -\sum_{j}p_{j}^{\left(0\right)}p_{j}^{\left(1\right)}\\ -\sum_{j}p_{j}^{\left(0\right)}p_{j}^{\left(1\right)} & \sum_{j}p_{j}^{\left(1\right)}\left(1-p_{j}^{\left(1\right)}\right) \end{array}\right)}^{-1}\left(v-\mu \right)$$

has asymptotic distribution $\chi^{2}$ with 2 degrees of freedom. But to use *T* with a known null distribution, it must be the case that the allele frequencies *π* for each SNP are known—and therefore ***F*** is known. We next show how to incorporate an estimated ***F*** into this statistic and determine computationally its null distribution. Note that when the null is true for unstructured HWE, the sums in the expression for Σ vanish, and *T* simplifies to the usual Pearson $\chi^{2}$ statistic, so our proposed statistic is a generalization of the usual $\chi^{2}$ test of HWE.

### Algorithm

In practice, the $\pi_{ij}$ values are unknown and the act of estimating them from the data being tested changes the null distribution of *T* (Chung and Storey 2015). We propose computing an empirical null distribution via the parametric bootstrap (Efron and Tibshirani 1993) by simulating data from the model of population structure, computing sHWE statistics for the simulated data set, and treating those statistics as samples from a null distribution. There are two aspects of the sHWE statistic that make the simulation of an empirical null attractive. First, models of population structure seek to account for the dependence present in data. Simulating an empirical null allows us to compute the sHWE statistic for data where the observed structure is preserved. Second, the statistic derived earlier is a pivotal quantity, *i.e.*, the null distribution is always $\chi^{2}$ with 2 degrees of freedom regardless of the values of $\pi_{ij}.$

Strictly speaking, each simulated data set serves as one bootstrap sample for each SNP. It would be too computationally intensive to simulate the data sets needed to have enough resolution to compute meaningful *P*-values. However, since the sHWE statistic is a pivotal quantity (so that each SNP has the same theoretical null distribution), pooling the simulated null data sets is an effective strategy. The pooling procedure is to simulate a small number of null data sets, and then combine the sHWE statistics for all of the simulated SNPs as observations from the null distribution. We validate the use of a pooled empirical null by comparing the distribution of *P*-values computed using the pooling routine and the distribution of marginal *P*-values, where an empirical null distribution is calculated for each SNP. We show quantile–quantile plots in Supplementary Material, Figure S1, between the *P*-values computed using the two types of null distributions. Their joint distributions are nearly identical, thus validating the pooled empirical null procedure (Leek and Storey 2011).

Our algorithm to test for sHWE in a data set of SNPs is described in Algorithm 1, which requires the user to select the number of null data sets *B* to generate to compute *P*-values. A graphical depiction of the algorithm is shown in Figure 2.

**Algorithm 1 Procedure for computing genome-wide sHWE P-values:** 

**Input**: A matrix of SNP genotypes ***X***, integer *B* for number of null data sets to generate.
**Output**: sHWE *P*-values.
**Initialization**: Form estimates ${\hat{\pi }}_{ij}$ from the genotype data ***X*** using a model and estimation method of population structure, such as LFA (Hao *et al.* 2016), truncated PCA (Hao *et al.* 2016), ADMIXTURE (Alexander *et al.* 2009), or TeraStructure (Gopalan *et al.* 2016).
**Observed statistics**: Compute sHWE statistics $T_{i}$ for each SNP i=1,…,m using ***X*** and ${\hat{\pi }}_{ij}$.
**for** b=1,…,B **do**
** Simulate null genotypes**: Create a null genotype matrix $X^{0}$ while preserving the observed population structure by drawing matrix elements $x_{ij}^{0}∼\text{Binomial}\left(2,{\hat{\pi }}_{ij}\right)$.
** Refit model of population structure**: Use the method for estimating ${\hat{\pi }}_{ij}$ on $X^{0}$ to compute estimates ${\hat{\pi }}_{ij}^{0}$.
** Null statistics**: Compute null sHWE statistics $T_{i}^{0}$ for each SNP i=1,…,m using $X^{0}$ and ${\hat{\pi }}_{ij}^{0}$.
**end**
**Compute *P*-values**: Pool the null statistics across all SNPs and simulated data sets to form an empirical null distribution. For each SNP *i*, compute *P*-values $p_{i}$ by
$p_{i}=\frac{\sum_{b=1}^{B}\sum_{k=1}^{m}1\left(T_{kb}^{0}\geq T_{i}\right)}{mB}$
where 1() is an indicator function.

### sHWE as empirical model tuning and validation

**Figure 3:**
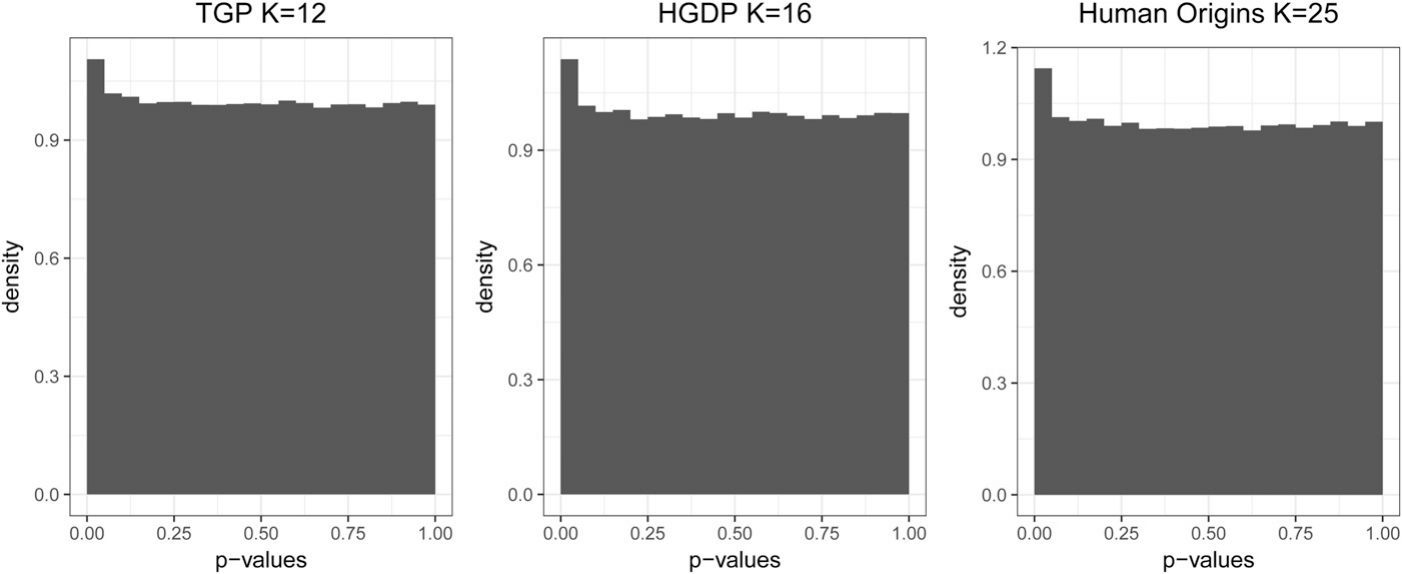
Histogram of sHWE test *P*-values for each data set at chosen *K* as determined by the entropy measure. The sHWE test is performed for each SNP in the data set after fitting the LFA model of population structure. The aggregated *P*-values are mostly Uniform(0, 1) distributed, except for a peak at 0. This indicates that most of the SNPs are in sHWE, given the fitted structure. The peak at 0 contains an enrichment of SNPs that deviate from sHWE.

Since many models of population structure parameterize $\pi_{ij},$ sHWE provides a framework for validating these models of population structure. By testing each individual SNP for violation of a model’s assumptions, we can aggregate the tests to determine if the overall population structure model accounts for the variation in the data appropriately. When the model is well formulated, the vast majority of SNPs should pass the sHWE test. Thus, we can examine the joint distribution of the *P*-values computed at every SNP in the data set. The expected behavior of the distribution of *P*-values is that the they are Uniform distributed across the interval [0,1], except near zero where a small portion of SNPs are shown to deviate from sHWE by having significant *P*-values (*e.g.*, Figure 3). Choosing the significance threshold can be done with a variety of methods, such as false discovery rates (FDRs) (Storey and Tibshirani 2003). This provides a natural criterion for filtering SNPs that violate the model assumptions and is an important part of any robust preliminary analysis.

This leads to a principled procedure for optimizing tuning parameters in the model of population structure such as the latent dimensionality *K*. If we compute sHWE *P*-values for a range of *K*, we can choose the value of *K* that has the best null properties. It is important to distinguish the characteristic of having good null properties from an absolute measure like least number of significant SNPs. Because our procedure is verifying a model fit over the genome, we want to choose the parametrization of $\pi_{ij}$ where the *P*-values are most Uniform distributed over the largest possible interval, excluding a possible peak near zero. The algorithm is detailed in Algorithm 2. This algorithm involves binning *P*-values into equal-sized bins to quantify how Uniform distributed they are over a given range. The number of bins is denoted by *C* in the algorithm; note that while we found to C=150 to be sufficient for analyses with 10^5^$\underset{\approx }{<}m\underset{\approx }{<}$10^6^, it may be helpful to choose a higher value if there are many more SNPs (or lower value for smaller data sets).

**Algorithm 2 Entropy-based procedure for automatically choosing the value of K:** 

**Input**: Genome-wide sHWE *P*-values over a range of *K*.
**Output**: Value of *K* with the best null properties of the *P*-values.
**for each** *K* **do**
** Bin *P*-values**: Divide the range of *P*-values into *C* equal-sized bins.
** Remove most significant bin**: Drop the bin with the lower bound of zero, since this bin should contain the most significant *P*-values.
** Compute proportions**: For each of the remaining C−1 bins, compute the proportion of *P*-values in each bin. These proportions should sum to one.
** Compute entropy**: Using these proportions, compute the entropy using the formula $-\sum_{c=2}^{C}f_{c}\text{log}\;f_{c}$ where $f_{c}$ is the proportion of *P*-values in the *c*-th bin.
**end**
**Identify optimal** *K*: Choose the value of *K* with the maximum entropy. In the event of a plateau where the entropy is more or less the same over a large range of *K*, then we suggest erring on the side of a smaller *K*, where the plateau begins. The plateau indicates that there is a range of *K* where the population structure model fits are similarly informative.

### Software

Our procedure is implemented in the lfa R package (Hao *et al.* 2016) (also available at http://github.com/StoreyLab/lfa) as the function sHWE().

### Data availability

The processed data sets are available at https://github.com/StoreyLab/sHWE-manuscript. Supplemental material available at Figshare: https://doi.org/10.25386/genetics.9876416.

## Results

We demonstrate the sHWE procedure on the global data sets detailed in *Methods: Data sets*. We also show that the sHWE procedure works with the truncated PCA method (Hao *et al.* 2016) and the ADMIXTURE method of fitting population structure (Alexander *et al.* 2009). Then, we consider a few ways to interpret the results of testing for sHWE. First, we show that there are no systematic differences in sHWE *P*-values when SNPs are separated by annotations or minor allele frequency. Then, we consider the replicability of sHWE results between the global data sets, as well as differences between results for the TGP samples on two different genotyping technologies.

### Analyzing global data sets

We demonstrate our proposed procedure where $\pi_{ij}$ is estimated using the LFA method (Hao *et al.* 2016) on three highly structured and global data sets: the HGDP, HO, and TGP (genotyping chip) data sets described in *Methods: Data sets*. We used B=3 null simulations from Algorithm 1 in the calculations. We show the sHWE *P*-value distributions over a range of *K*, the latent dimensionality of the LFA model of population structure, for the three data sets in Figures S2, S3, and S4. The distributions of *P*-values share the same general behavior between data sets. When *K* is too small and the population structure is insufficiently modeled, the sHWE test *P*-values are skewed heavily toward zero. As additional latent factors are added to account for more structure across the genome, the *P*-value distributions shift away from zero and become more Uniform. Eventually, the *P*-value distributions become skewed toward one, as population structure model is overfit to the data.

**Figure 4:**
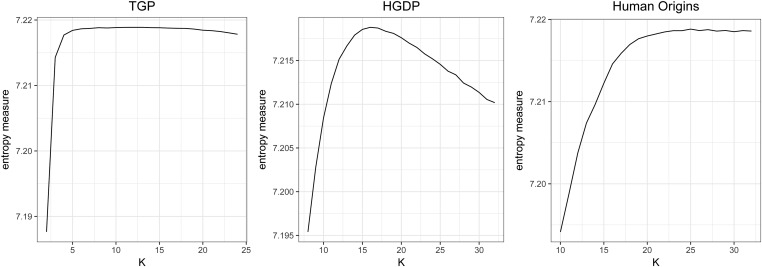
The entropy measure of uniformity of *P*-values for each data set as a function of *K*. For each model fit and value of *K*, the *P*-values for each data set were summarized by counting the number in each of 150 equal-sized bins in the range [0,1]. The bin closest to zero was dropped, as the most significant *P*-values will be in that bin. The proportion of counts in the 149 bins remaining are used to compute the entropy corresponding to *K*. Higher entropy means more Uniform.

For each data set, we observe that there is a range of *K* where we observe the desired distribution of *P*-values, *i.e.*, a peak near zero and Uniform elsewhere. Model fits in this range of *K* have the highest confidence of being well formulated and all serve equally well as a basis for future analysis. We suggest choosing *K* following the entropy measure presented in *Methods: sHWE as empirical model tuning and validation*. We show results in Figure 4, corresponding to K=12 for TGP, K=16 for HGDP, and K=25 for HO. At these values of *K*, we estimate the proportion of SNPs that are in sHWE, using the bootstrap method from the qvalue R package (Storey and Tibshirani 2003). We find these estimated proportions to be 0.990 for TGP, 0.990 for HGDP, and $\pi_{0}=0.989$ for HO; this suggests that the vast majority of human SNPs are in HWE. SNPs in sHWE are also interpreted to be well parameterized by the LFA population structure model.

To demonstrate results using other parameterizations of $\pi_{ij}$, we first analyze these three data sets for a range of *K* using the truncated PCA method (Hao *et al.* 2016). The resulting histograms (Figures S5, S6, and S7) are comparable with those estimated using $\pi_{ij}$, except that there is a small peak near one for larger values of *K*. This likely reflects noise introduced by the truncation procedure.

We also tested parametric models of population structure. We used the ADMIXTURE method (Alexander *et al.* 2009), which is a widely used software for fitting the admixture model of population structure, before applying the sHWE procedure. The resulting figures for the HO data set are shown in Figures S8 and S9. The sHWE *P*-values exhibit the expected behavior in terms of histogram shape over the range of *K*. We note that the entropy measure plateaus at K=30, which is a higher value of *K* than with the LFA method on this data set. This is expected behavior, as the admixture model is more constrained than the LFA model, since the factors need to be valid probabilities. Thus, a higher *K* is needed to achieve a similar fidelity in the modeled population structure. Further, while the sHWE procedure works with ADMIXTURE, it is a more arduous task computationally, since the individual model fits are slower than with LFA, and the sHWE procedure requires multiple fits per value of *K*.

### The role of SNP annotation in deviations from sHWE

We compared the distributions of sHWE *P*-values in each data set when separated by functional annotations of the SNPs (Hinds 2005). We considered three nested levels of labels. First, SNPs were separated into intragenic and intergenic categories. Then, the intergenic SNPs were separated by whether they were in an exon or an intron. Lastly, the exonic SNPs were separated into synonymous and nonsynonymous mutations. For each data set, we found no differences between the distributions of sHWE *P*-values for each of the categories (Figures S10, S11, and S12). Further, we found minimal differences in the distribution of *P*-values when binned by minor allele frequency (Figures S13, S14, and S16).

### Replicability of sHWE between data sets

**Figure 5:**
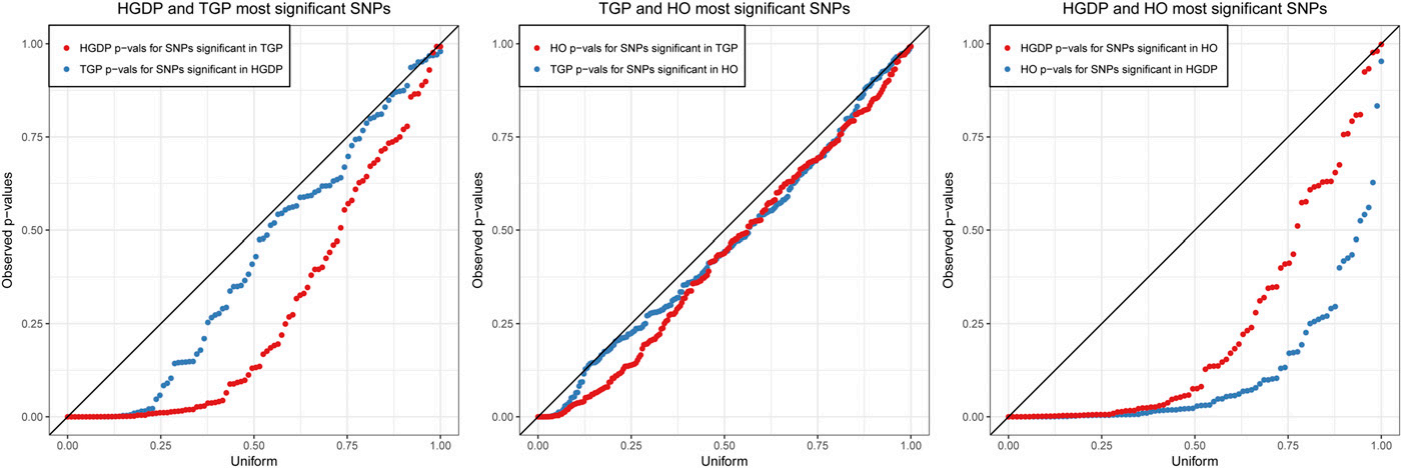
Comparisons of significant sHWE *P*-values between the three data sets. For each pair of data sets, we choose the *S* most significant SNPs from one data set, where *S* is the greater of the number of significant SNPs at FDR *q*-value ≤ 20% for both data sets. We then test the corresponding *S* SNPs for sHWE in the other data set. Quantile–quantile plots of the resulting *P*-values *vs.* the Uniform(0, 1) quantiles shows that the deviations from sHWE are enriched in the other data set, verifying concordance of departures from sHWE between data sets.

To demonstrate the robustness of our sHWE procedure, we compared the results between data sets by analyzing the overlapping SNPs. For each pair of data sets, we first identified the SNPs shared by the two data sets. Between HGDP and TGP there were 357,314 shared SNPs, between HO and HGDP there were 130,572 shared SNPs, and between TGP and HO there were 163,443 shared SNPs. Within each of the three pairs of data sets, we compared the two sets of shared *P*-values by examining the most significant tail of the distribution of *P*-values. We chose the length of the tail by identifying how many SNPs are significant within each of the six sets of *P*-values at a 20% FDR threshold using the qvalue R package (Storey and Tibshirani 2003). Then, for each pair, we chose the larger number of significant SNPs. The goal of this approach was to choose enough SNPs such that we capture a reasonable number of significant and nonsignificant SNPs. We observed concordance between the data sets because SNPs that were significant in one data set showed sHWE *P*-values in the other data set that are skewed toward zero and stochastically less than the Uniform(0, 1) distribution. If there were no concordance we would expect these replication *P*-values to be approximately Uniform(0, 1), which they are not. This suggests that deviations from sHWE show concordance between data sets, which in turn suggests that some of the effects driving the violation of sHWE are shared between data sets (*i.e.*, biological) and not unique to a data set (*i.e.*, genotyping errors). The similarity between data sets is strongest in the comparison between the HGDP and HO data sets, which also share many of the same individuals, albeit genotyped on different technologies. These comparisons are shown in Figure 5.

### Linkage disequilibrium

To assess the impact of linkage disequilibrium on our sHWE procedure, we generated data sets based on the TGP genotyping array data set using varying thresholds for minimum distance between SNPs. The original data set had no threshold, so SNPs could be arbitrarily close. We generated two data sets enforcing SNPs to be at least 1 kbp apart and 10 kbp apart, respectively, and then we carried out the sHWE analysis as above on each data set. We compared the sHWE *P*-value distributions of both of these data sets with the original data set in Figure S15 by using a quantile–quantile plot of sHWE *P*-values computed on all SNPs in each data set. We observed no difference in distributions in either case. As with any analysis of genome-wide genotyping data, *P*-values of SNPs in linkage disequilibrium will be dependent, so this should be taken into account when assessing joint statistical significance.

### sHWE between genotyping technologies

The TGP provides a controlled setting to further investigate sHWE in genome-wide data because samples have been genotyped using different technologies. In addition to genotyping chip data, we also incorporated the integrated variant callset made by the TGP, which are derived primarily from sequencing data. We created a subset of both the genotype chip data and integrated callset that share the exact same individuals while maintaining a maximal overlap in the SNPs (see *Methods*). We then calculated sHWE *P*-values for both data sets at K=12, which was determined earlier for the TGP data set.

**Figure 6:**
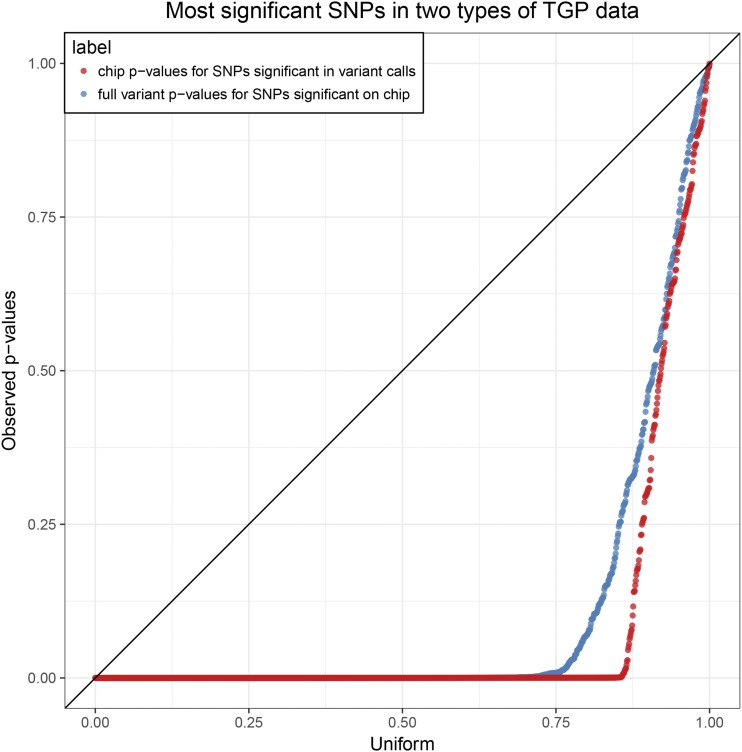
Comparisons of sHWE *P*-values between TGP genotyping array data and the TGP variant calls. We identify significant SNPs at FDR *q*-value ≤ 20% for the two data sets, then plot quantile–quantile plots of the SNPs shared in the other data set against the Uniform distribution. The labelings follow the convention in Figure 5.

To compare the results between the technologies, we employed two approaches. First, we analyzed the shared SNPs between the data sets generated using an approach identical to the between-data sets comparison earlier, using maximum number of significant SNPs at FDR *q*-value ≤ 20% (Figure 6). We observe that the majority of sHWE *P*-values in one data set for SNPs that are significant in the other are extremely small, although they are not necessarily significant at this particular significance threshold. This still represents concordance as the *P*-values in the other data set are stochastically much smaller than Uniform. The remaining *P*-values are linear, meaning that the right tail of the *P*-values are approximately Uniform away from zero. This shows that the majority of SNPs (∼75–80%) that deviated from sHWE exhibit this behavior in both data sets.

Then, we compared the distributions of all sHWE *P*-values between the two data sets for the shared and unshared SNPs (Figure S17). While the distribution of sHWE *P*-values for all shared SNPs is nearly identical, there are proportionally many more significant SNPs deviated from HWE in the integrated variant callset than in the genotyping chip data. This suggests that SNPs called from the sequencing data are less accurate than those genotyped using chips.

## Discussion

We extended the Pearson $\chi^{2}$ test of HWE to allow for population structure, called the sHWE test. This allows one to identify genetic markers that deviate from HWE for reasons other than population structure. For example, SNP markers can be identified in a GWAS with structure that potentially have genotyping errors, or genetic loci that are subject to evolutionary forces of interest other than structure can be identified for further analysis.

Our proposed approach is flexible in terms of the exact formulation of the model of structure. It only requires that each SNP and individual pair is drawn from a Binomial distribution, which is a condition satisfied by most common models of population structure. We chose to employ the LFA model here (Hao *et al.* 2016), which serves as a base model of population structure for a test of association in GWAS (Song *et al.* 2015). A caveat of our sHWE procedure is that simulating the empirical null distribution means that we are reliant on computationally efficient methods for modeling population structure.

We demonstrated the proposed sHWE test on three highly structured global data sets. In each data set, we showed there is a configuration of the population structure model that captures the full range of genetic variation for ∼99% of the SNPs and that the testing procedure provides a metric for choosing the dimension.

Model validation is an important preliminary step when applying probabilistic models to genome-wide genotyping data. We have shown that our sHWE test is a powerful tool for doing so. This approach to goodness of fit is applicable to any high-dimensional latent structure model for which it is possible to efficiently simulate data from a given model fit. Further, our sHWE procedure yields the ability to examine a wider range of biological questions, as our understanding of deviations from HWE in unstructured populations can now be applied to structured populations.
